# Altered effective connectivity in leucine-rich glioma-inactivated 1 antibody encephalitis: a spectral dynamic causal modeling study

**DOI:** 10.1093/psyrad/kkaf022

**Published:** 2025-08-20

**Authors:** Jianping Qiao, Lele Zheng, Wenlong Xu, Xuefeng Zang, Hao Shang, Cuicui Li, Shengjun Wang, Anning Li

**Affiliations:** School of Physics and Electronics, Shandong Normal University, Jinan, 250014, China; School of Physics and Electronics, Shandong Normal University, Jinan, 250014, China; School of Physics and Electronics, Shandong Normal University, Jinan, 250014, China; School of Physics and Electronics, Shandong Normal University, Jinan, 250014, China; School of Physics and Electronics, Shandong Normal University, Jinan, 250014, China; Department of Radiology, Shandong Provincial Hospital Affiliated to Shandong First Medical University, Jinan, 250117, China; Department of Neurology, Qilu Hospital, Cheeloo College of Medicine, Shandong University, Jinan, 250012, China; Department of Radiology, Qilu Hospital, Cheeloo College of Medicine, Shandong University, Jinan, 250012, China

**Keywords:** anti-LGI1 encephalitis, fMRI, effective connectivity, spectral dynamic causal modeling

## Abstract

**Background:**

Despite advances in understanding the effective connectivity (EC) of brain networks in leucine-rich glioma-inactivated 1 (LGI1) antibody encephalitis, the specific cause and underlying mechanisms of LGI1 encephalitis remain unclear.

**Materials and methods:**

The study included 27 patients with anti-LGI1 encephalitis and 28 age- and sex-matched normal controls. Amplitude of low-frequency fluctuation (ALFF) analysis identified altered brain regions. Spectral dynamic causal modeling (spDCM) then assessed EC between these regions. Relationships between EC strength and both clinical severity and cognitive function were analyzed.

**Results:**

Distinct EC patterns were found in patients versus controls. Specifically, inhibitory EC was observed from the hippocampus to the superior temporal gyrus, while excitatory EC was noted in the reverse direction. Patients also showed reduced inhibitory self-connections in the posterior cingulate cortex. Crucially, inhibitory EC from the right hippocampus to the left superior temporal gyrus correlated inversely with symptom severity and positively with cognitive performance. Conversely, reduced inhibitory self-connections in the posterior cingulate cortex correlated positively with symptom severity and negatively with cognitive function.

**Conclusions:**

These findings indicate that changes in causal connections between specific brain regions significantly contribute to neurological deficits in anti-LGI1 encephalitis. The inhibitory connectivity from the hippocampus to the superior temporal gyrus may serve as a potential biomarker for personalized diagnosis, offering new insights into the underlying pathological mechanisms of this disorder.

## Introduction

Leucine-rich glioma inactivated 1 (LGI1) antibody encephalitis is an autoimmune disorder characterized by autoantibody-mediated neurological dysfunction. Its clinical features include seizures particularly facial and brachial dystonic seizures (FBDS), memory impairment, sleep disturbances, altered consciousness, and hyponatremia (Huang *et al*., 2022). Notably, FBDS, characterized by abnormal muscle contractions in the unilateral face and extremity (arm or leg), often precedes an anti-LGI1 encephalitis diagnosis (Irani *et al*., 2011).

Currently, the definitive diagnosis of LGI1 encephalitis relies on detecting LGI1 antibodies in serum or cerebrospinal fluid through a blood draw or lumbar puncture. Neuroimaging techniques could serve as a non-invasive diagnostic method. However, they lack specific biomarkers for this disorder, and more than half of patients may not exhibit structural abnormalities (Heine *et al*., 2018). Investigating brain functional alterations in LGI1 encephalitis patients using non-invasive imaging techniques is crucial for elucidating the underlying pathophysiological mechanisms and opens new avenues for the use of neuroimaging to identify potential biomarkers for diagnosis and treatment.

Neuroimaging techniques have revealed significant functional and structural abnormalities in brain regions that are implicated in emotions, memory, and seizures in patients with anti-LGI1 encephalitis, including the medial temporal lobe, amygdala, hippocampus, cingulate cortex, and insula (Finke *et al*., 2017; Hang et al., 2020). Prior positron emission tomography (PET) studies have identified heightened basal ganglia abnormalities and severe metabolic hypoplasia associated with FBDS in individuals with autoantibodies against LGI1 (Flanagan *et al*., 2015; Burkett *et al*., 2023). Structural magnetic resonance imaging (MRI) studies have demonstrated reduced volumes in the hippocampus, corpus callosum, dentate gyrus, and pallidum among LGI1 encephalitis patients (Finke *et al*., 2017). Furthermore, diffusion tensor imaging (DTI) has shown impaired microstructural integrity in the hippocampus, corona radiata, internal capsule, and corpus callosum in anti-LGI1 encephalitis patients (Szots *et al*., 2017; Gatto *et al*., 2018).

Magnetic resonance spectroscopy (MRS) analysis has also revealed decreased glutamine levels and glutamatergic white matter in patient groups compared to normal controls (Szots *et al*., 2017). These findings suggest that the widespread structural and microstructural changes observed in anti-LGI1 encephalitis are linked to its clinical manifestations and provide insights into the pathological mechanisms underlying this disease.

Functional MRI (fMRI) studies have revealed alterations in brain function in anti-LGI1 encephalitis patients, using functional connectivity to evaluate brain networks (Finke *et al*., 2017; Nantes *et al*., 2018). These studies have consistently shown reduced functional connectivity in memory, cognition, and motor circuits among anti-LGI1 encephalitis patients (Qiao *et al*., 2020). Furthermore, disruptions in the default mode network (DMN), and sensorimotor, salience, and visual networks have been reported (Nantes *et al*., 2018). The amplitude of low-frequency fluctuations (ALFF) quantifies the magnitude of low-frequency oscillations in resting-state blood oxygen level-dependent (BOLD) signals, representing periodic shifts in macroscopic cortical excitability and interregional neuronal coherence (Zou *et al*., 2008). It has been proved relative to spontaneous brain activity in many brain diseases (Gong *et al*., 2020). An analysis of ALFF revealed decreased low-frequency oscillations in the hippocampus of patients compared to controls, underscoring functional changes that may underlie clinical symptoms and corroborate structural findings.

However, functional connectivity alone, which detects undirected connections between brain regions, fails to capture the directional flow of information. Consequently, the causal influences underpinning brain circuits in anti-LGI1 encephalitis remain elusive. Effective connectivity, which quantifies causal relationships and inhibitory/excitatory influences between regions, is crucial for elucidating functional integration in this disease. Dynamic causal modeling (DCM), particularly spectral DCM (spDCM) for resting-state fMRI, offers a model-based approach to identify causal and directional connections, demonstrating greater sensitivity to group differences compared to stochastic DCM (Friston *et al*., 2003, 2014, 2014; Razi *et al*., 2015).

In this study, we examined resting-state effective connectivity alterations in LGI1 antibody encephalitis and their potential associations with clinical disease severity and cognitive function by utilizing fMRI data. We employed ALFF-based multivoxel analysis and two-sample t-tests to pinpoint distinct brain regions exhibiting significant differences between anti-LGI1 encephalitis patients and healthy controls. Subsequently, spDCM was used to shed light on the causal relationships and directional connections between these identified regions. We hypothesized that disruptions in directed or effective connectivity within intrinsic brain networks contribute to memory, cognition, and motor impairments in anti-LGI1 encephalitis patients.

## Materials and methods

### Participants

This study included 27 patients (19 males, eight females; mean age 55 ± 14 years) with anti-LGI1 encephalitis, recruited from Qilu Hospital of Shandong University. All patients were diagnosed through serum LGI1 antibody testing, with antibody titers mainly at 1:10, 1:32, and 1:100. We designated a low antibody titer of 1:10 as (+) score, and titers of 1:32 and 1:100 as (++) score. One patient received a (+) score, while 26 patients had a (++) score or higher. All patients were diagnosed via the detection of serum LGI1 antibodies, with one patient exhibiting a (+) score and 26 patients a (++) score. The modified Rankin Scale (mRS) was utilized to evaluate patient symptom severity. Cerebrospinal fluid (CSF) examinations were performed on 24 patients, revealing normal white blood cell counts in 16 patients and elevated protein concentrations in eight (range: 0.46–1.47 g/L; normal <0.45 g/L). Additionally, lactate concentrations were found to be elevated in eight patients (range: 2.3–2.9 mmol/L; normal 1.2–2.1 mmol/L). Cognitive function was assessed using the Mini-Mental State Examination (MMSE) and the Montreal Cognitive Assessment (MoCA).

Table 1 summarizes the demographic and clinical characteristics of the patients. For comparison, 28 normal controls (19 males, nine females; mean age 52.5 ± 10.4 years), matched by gender and age, were recruited. All participants provided written informed consent, and the study protocol was approved by the Institutional Review Board of Qilu Hospital of Shandong University.

**Table 1:** Demographic and clinical characteristics of anti-LGI1 encephalitis patients.

Characteristics	No.
Age, mean (SD)	55 (14.28) years
Sex	19 male, 8 female
Modified Rankin Scale score^a^, mean (SD)	2.24 (0.77)
Mini-Mental State Examination (MMSE), mean (SD)	19.67 (6.94)
Montreal Cognitive Assessment (MoCA), mean(SD)	16.19 (6.95)
**Symptom**	
Memory impairment	21/27 (78%)
Seizure	18/27 (67%)
Faciobrachial dystonic seizures	11/27 (41%)
**Cerebrospinal fluid**	
Protein^b^ (g/L), mean (SD)	0.42 (0.29)
Lactic acid^c^ (mmol/L), mean (SD)	2.07 (0.66)
**Antibodies to LGI1**	
Serum (positive)	27/27 (100%)
Cerebrospinal fluid (positive)	11/24 (46%)

a The criteria of the modified Rankin Scale (mRS) score can be described as: 0—no symptoms at all; 1—no significant disability despite symptoms: able to carry out all usual duties and activities; 2—slight disability: unable to carry out all previous activities, but able to look after own affairs without assistance; 3—moderate disability: requiring some help, but able to walk without assistance; 4—moderately severe disability: unable to walk and attend to bodily needs without assistance; 5—severe disability: bedridden, incontinent and requiring constant nursing care and attention. ^b^CSF protein normal values: <0.45 g/L. ^c^CSF lactic acid normal values: 1.2–2.1 mmol/L.

### Data acquisition

The resting-state fMRI data for all participants were acquired using a Siemens Verio 3.0T MRI scanner equipped with a 32-channel head coil (Siemens Healthcare Sector, Germany) at Qilu Hospital of Shandong University. During the scan, the patients were instructed to close their eyes and remain awake in a supine position. Foam pads were used to immobilize the head and body to minimize head movement artifacts. The fMRI data were collected using a single gradient-echo echo-planar imaging (EPI) sequence with the following parameters: repetition time (TR) = 2000 ms, echo time (TE) = 30 ms, flip angle = 90°, field of view (FOV) = 24 × 24 cm, matrix size = 64 × 64, voxel size = 3.4 × 3.4 × 4.0 mm³, and slice thickness = 3 mm. The total duration of data collection was approximately 8 min.

### Image preprocessing

**Figure 1:**
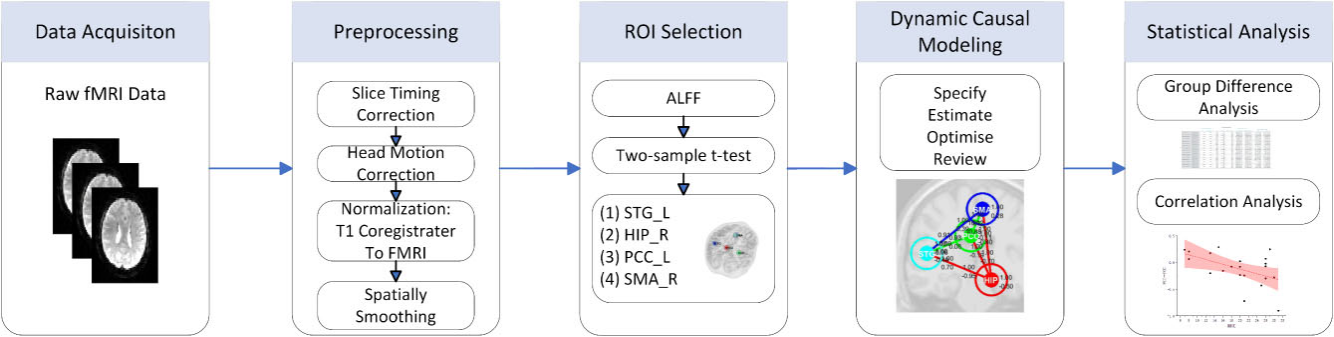
Flowchart of the effective connectivity analysis.

The effective connectivity within the brain was investigated using the spDCM approach on the resting-state fMRI data. This process encompassed four key steps: image preprocessing, region of interest (ROI) selection, DCM modeling, and statistical analysis. A flowchart illustrating the methodology is presented in Fig. 1. The fMRI data underwent extensive preprocessing using the DPABI toolbox (Yan *et al*., 2016) (http://www.rfmri.org/dpabi), which is built upon the SPM12 software (http://www.fil.ion.ucl.ac.uk/spm).

The first 10 slices were discarded to ensure signal stabilization. Then, slice timing correction was applied to account for the interleaved slice acquisition in the functional data. Motion correction was subsequently performed to mitigate the effects of head movement. Subjects were excluded by head motion of >2 mm in maximum displacement or >2° rotation in angular motion. The corrected functional images were then aligned to the bias-corrected T1-weighted structural images. Tissue segmentation was conducted, segregating the transformed structural images into gray matter, white matter, and cerebrospinal fluid, generating differential anatomical alignment templates. All images were then normalized to the Montreal Neurological Institute (MNI) standard spatial coordinate system using the DARTEL template. Finally, spatial smoothing was applied using a 4 × 4 × 4 mm Gaussian kernel with a full width at half maximum to enhance the signal-to-noise ratio.

### Identification of regions of interest

We used the ALFF method with statistical analysis to identify ROIs in the preprocessed fMRI data of healthy controls and LGI1 encephalitis patients. Specifically, we calculated ALFF values based on the power spectra of the time series data, focusing on a low-frequency range of 0.01–0.08 Hz. These values represented the regional spontaneous activity intensity in the BOLD signals. Last, a two-sample t-test was performed on the ALFF values to identify regions exhibiting significant differences between the healthy controls and LGI1 encephalitis patients (uncorrected *P* < 0.01).

### Effective connectivity analysis

spDCM is an advanced analysis method that leverages endogenous neural fluctuations in the frequency domain to fit complex cross-spectral densities. For spDCM, considering *z* as a single state brain activity in some certain ROIs, a neural state equation and observation equation in Langevin form can be defined as follows:


(1) \begin{eqnarray*}
\dot{z} &=& f\left( {z,u,\theta } \right) + v\\
y &=& f\left( {z,u,\phi } \right) + e
\end{eqnarray*}

where ${\mathrm{\dot{z}}}$ is the rate in change of the neuronal states *z, f*( · ) is a non-linear function, *u* is exogenous inputs and usually set to zero in the resting state, θ is the parameter of the model (i.e. the effective connectivity), and *v* is the stochastic process—called the state noise (respectively the measurement or observation noise). This equation can be represented in simple linear form as:


(2) \begin{eqnarray*}
\dot{z} = Az + Cu + v
\end{eqnarray*}

where *A* is the Jacobian describing the effective connectivity.

Nevertheless, these two models both require us to estimate hidden states, which poses a difficult inverse problem that is computationally demanding. To solve the problem, spDCM estimates the time-invariant parameters of ROI cross-spectra, which means second-order statistics need to be calculated. The method is as follows:


(3) \begin{eqnarray*}
{g}_{v}(\omega ,\theta) &=& {\alpha}_{v}{{\omega }^{ - {{\beta }_v}}}\\
{{g}_e}(\omega ,\theta) &=& {{\alpha }_e}{{\omega }^{ - {{\beta }_e}}}
\end{eqnarray*}

where {α, β}⊂θ. Using the model parameters, θ⊆{*A, C*, α, β}, we can simply generate the expected cross-spectra:


(4) \begin{eqnarray*}
y &=& \boldsymbol{\mathcal{K}}*v + e\\
\boldsymbol{\mathcal{K}} &=& {{\partial }_z}h\exp \left( {t{{\partial }_z}f} \right)\\
{g}_{y}(\omega ,\theta) &=& {{\left| {K\left( \omega \right)} \right|}^2}{{g}_v}(\omega ,\theta) + {{g}_e}(\omega ,\theta)
\end{eqnarray*}

In this study, we employed the DCM12 module within SPM12 to perform spDCM.

Initially, a generalized linear model was applied to eliminate noise from white matter and cerebrospinal fluid. Subsequently, mean time series were extracted from the ROIs within an 8-mm sphere. A full connectivity model was then conducted for each subject to identify bidirectional connections between ROIs, excluding exogenous inputs (Friston *et al*., 2014). A previous study (Qiao *et al*., 2020) demonstrated that LGI1 antibody encephalitis exhibited connectivity changes in hippocampus, inferior frontal gyrus, amygdala, and supplementary motor area, among others. We therefore selected four most privileged ROIs for further analysis. With four ROIs, this process resulted in 16 free parameters for effective ROI-to-ROI connections. As spDCM operates in the frequency domain, coupling parameters were fitted using second-order statistics to characterize the spectral density. Bayesian model selection (BMS) was then employed to identify the best-fitting model, balancing computational accuracy and complexity.

To detect conserved effective connectivity across subjects within each group, a second-level parametric analysis was conducted using a parametric empirical Bayesian (PEB) model. The model parameters were estimated using the standard variational Laplacian, with the posterior probability (PP) rather than traditional *P*-values used to highlight group differences. *Post hoc* BMS was then performed on the full model with all free parameters (Friston and Penny, 2011), enabling the identification of the most suitable model parameters for each group within the spDCM framework. When there were more than 16 free parameters, BMS efficiently conducted a greedy search through all permutations of eight parameters, resulting in 256 simplified models (Rosa *et al*., 2012). The simplified model with the highest model posterior probability was selected as the best-fit one for each group.

### Statistical analyses

We used a two-sample t-test in SPSS 26.0 to determine significant differences in valid connectivity measures between patients and controls. Prior to statistical analyses, we tested the data for normality, selecting setups for analysis, and we also performed Pearson correlation analyses and Spearman correlation analyses to assess the strength and validity of the relationships between clinical indicators (mRS, MMSE, and MOCA scores) and measures of directed connectivity.

## Results

### Detected ROIs

**Figure 2:**
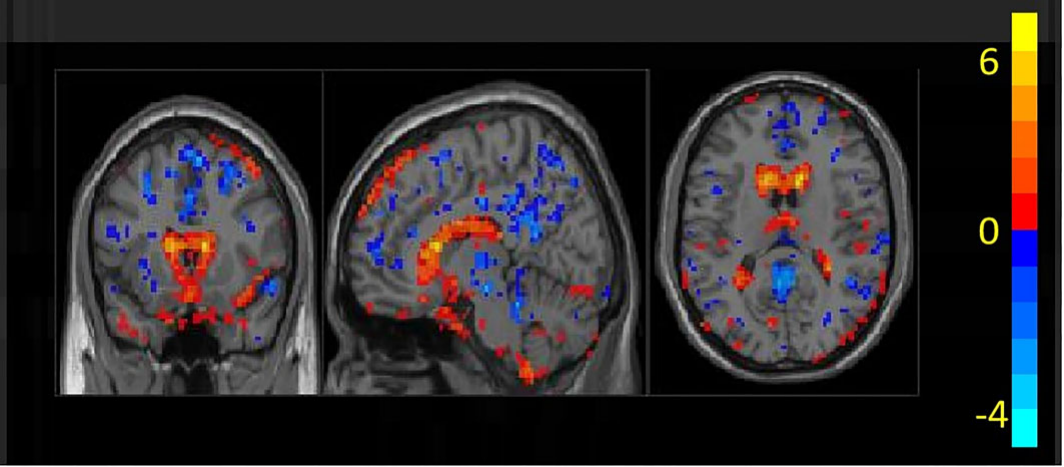
Difference of the ALFF between groups.

The difference of ALFF between LGI1 encephalitis patients and normal controls is shown in Fig. 2. We identified four significant regions: the hippocampus, posterior cingulate cortex, supplementary motor area, and superior temporal gyrus by using a two-sample t-test with a significance threshold of *P* < 0.001. The precise locations of these ROIs are shown in Table 2.

**Table 2:** Specific locations of ROIs with the two-sample t-test of ALFF values between patients and controls.

		Peak MNI (mm)			
Brain region	Hemisphere	X	Y	Z	Voxels
Hippocampus	R	18	−9	−15	77
Posterior cingulate cortex	L	−3	−45	30	134
Supplementary motor area	R	9	−12	57	62
Superior temporal gyrus	L	−48	−27	12	90

### Within-group effects in anti-LGI1 encephalitis

**Figure 3:**
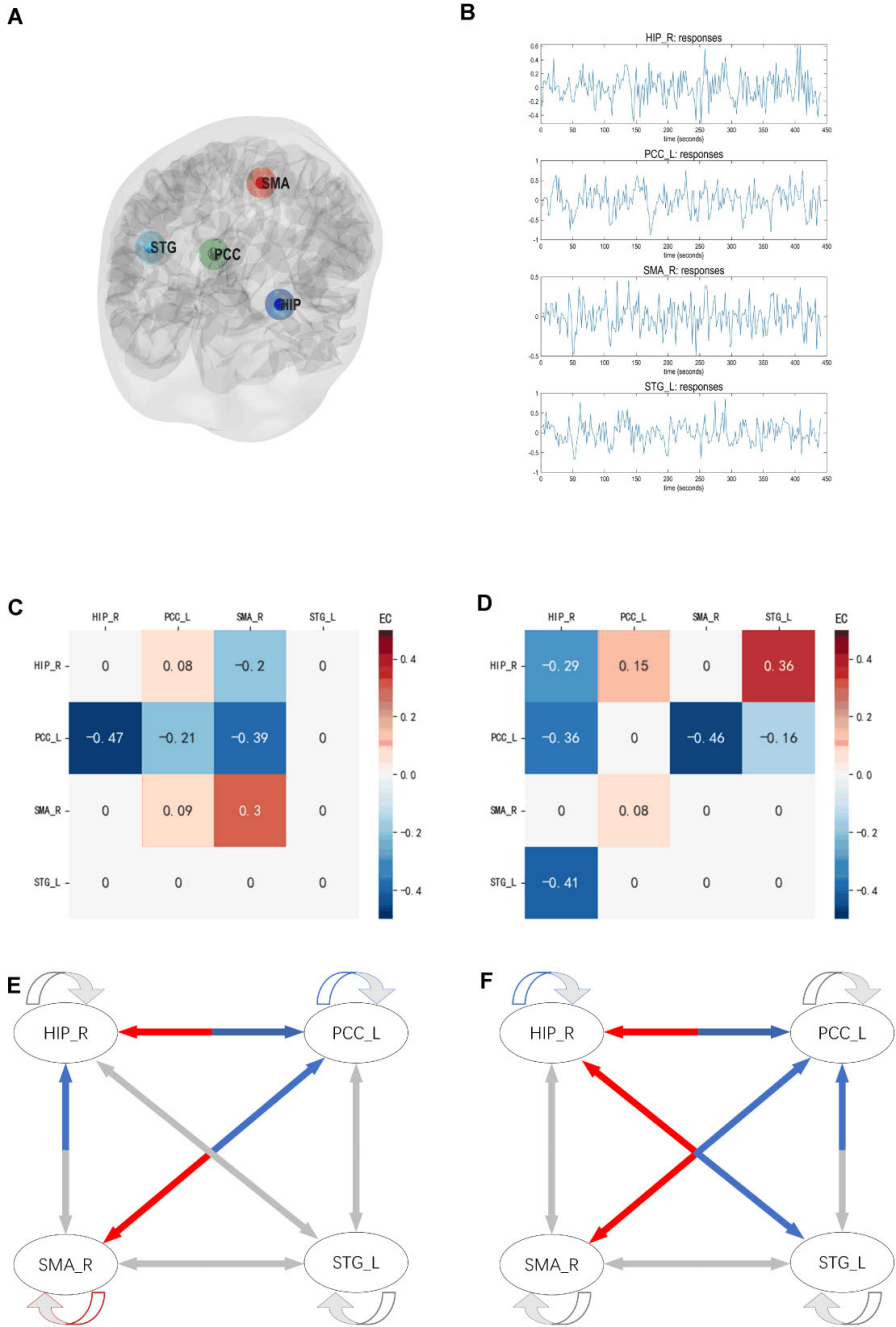
Within-group effects in anti-LGI1 encephalitis patients and controls. (**A**) Locations of the four regions of interest (ROIs) were used for spectral dynamic causal modeling (spDCM) analysis. (**B**) The time series of ROIs in an anti-LGI1 encephalitis patient were used to invert spDCM with a fully connected structure. (**C**) spDCM results obtained from the control group. (**D**) spDCM results obtained from the LGI1 patient group. (**E**) Effective connectivity (EC_ plots of the control group) (*P* < 0.01). (**F**) EC plots of the LGI1 patient group (*P* < 0.01). Warm colors indicate high parameter estimation, and cool colors indicate low parameter estimation. Abbreviations: L: left, R: right, HIP: hippocampus, PCC: posterior cingulate cortex, SMA: supplementary motor area, STG: superior temporal gyrus, EC: effective connectivity.

The PEB algorithm estimated 16 free parameters within the DCM model, characterizing the effective connectivity between the four identified ROIs (hippocampus, posterior cingulate cortex, supplementary motor area, and superior temporal gyrus) and their self-connectivity, for both controls and patients. Figure 3 illustrates the significant effective connectivity within each group at *P* < 0.01, and Table 3 demonstrates the effective connectivity calculated by the DCM model. Compared to controls, patients exhibited inhibitory connections from the right hippocampus to the posterior cingulate cortex and superior temporal gyrus, and inhibitory effects from the supplementary motor area to the posterior cingulate cortex, and the superior temporal gyrus back to the posterior cingulate cortex. Additionally, patients showed excitatory connections from the posterior cingulate cortex to the hippocampus, supplementary motor area, and the superior temporal gyrus back to the hippocampus (all PP > 0.99).

**Table 3:** Normality testing of EC and clinical data.

		Shapiro-Wilkes		
		Statistic	df	Significance
NC	HIP(R) vs HIP(R)	0.965	28	0.465
	PCC(L) vs HIP(R)	0.805	28	0
	SMA(R) vs HIP(R)	0.972	28	0.627
	STG(L) vs HIP(R)	0.958	28	0.314
	HIP(R) vs PCC(L)	0.965	28	0.443
	PCC(L) vs PCC(L)	0.966	28	0.472
	SMA(R) vs PCC(L)	0.982	28	0.891
	STG(L) vs PCC(L)	0.873	28	0.003
	HIP(R) vs SMA(R)	0.966	28	0.482
	PCC(L) vs SMA(R)	0.799	28	0
	SMA(R) vs SMA(R)	0.948	28	0.173
	STG(L) vs SMA(R)	0.906	28	0.016
	HIP(R) vs STG(L)	0.934	28	0.078
	PCC(L) vs STG(L)	0.769	28	0
	SMA(R) vs STG(L)	0.914	28	0.025
	STG(L) vs STG(L)	0.972	28	0.631
PT	HIP(R) vs HIP(R)	0.963	27	0.437
	PCC(L) vs HIP(R)	0.919	27	0.038
	SMA(R) vs HIP(R)	0.94	27	0.124
	STG(L) vs HIP(R)	0.983	27	0.923
	HIP(R) vs PCC(L)	0.916	27	0.031
	PCC(L) vs PCC(L)	0.923	27	0.047
	SMA(R) vs PCC(L)	0.976	27	0.755
	STG(L) vs PCC(L)	0.952	27	0.238
	HIP(R) vs SMA(R)	0.968	27	0.546
	PCC(L) vs SMA(R)	0.977	27	0.792
	SMA(R) vs SMA(R)	0.94	27	0.12
	STG(L) vs SMA(R)	0.945	27	0.162
	HIP(R) vs STG(L)	0.948	27	0.188
	PCC(L) vs STG(L)	0.976	27	0.776
	SMA(R) vs STG(L)	0.969	27	0.577
	STG(L) vs STG(L)	0.982	27	0.912
Scores	mRS	0.791	27	0
	MMSE	0.919	27	0.082
	MOCA	0.962	27	0.561

R: right, L: left, HIP: hippocampus, PCC: posterior cingulate cortex, SMA: supplementary motor area, STG: superior temporal gyrus, mRS: modified Rankin Scale, MMSE:Mini-Mental State Examination, MOCA: Montreal Cognitive Assessment.

Notably, the connection from the superior temporal gyrus to the hippocampus was weaker in controls but significantly enhanced in patients, whereas the connection from the hippocampus to the superior temporal gyrus was inhibitory in patients compared to controls.

### Effective connectivity alterations in anti-LGI1 encephalitis

**Figure 4.**
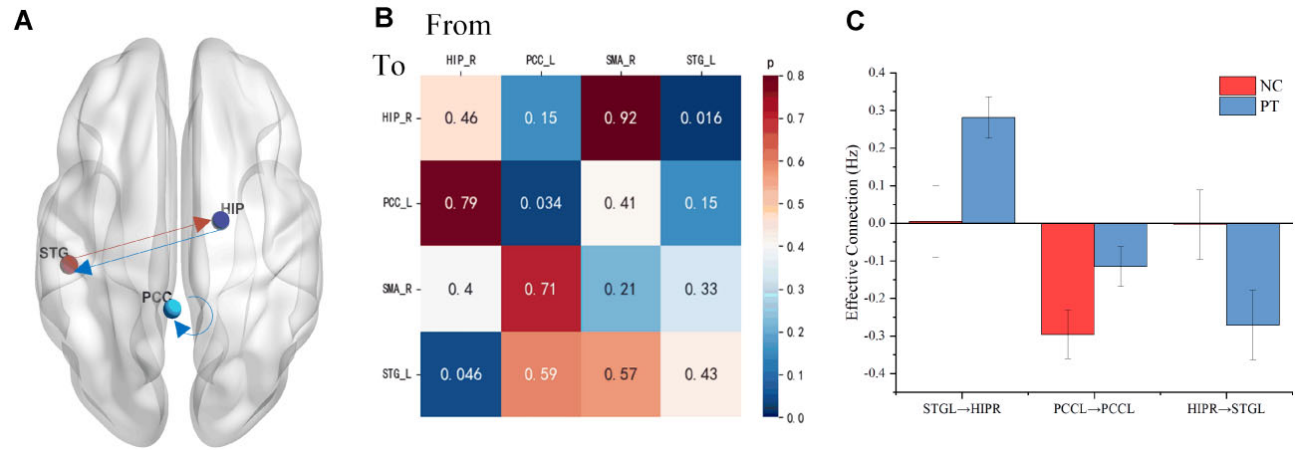
Group differences between anti-LGI1 encephalitis patients and controls. (**A**) Comparison of effective connectivity strength between the groups. (**B**) Two independent samples t-test results. (**C**) Mean effective connectivity of significantly altered regions.

Figure 4 shows significant differences in the strength of effective connectivity between patients and controls. Notably, we observed significant variations in bidirectional connectivity between the hippocampus and superior temporal gyrus and variation in the self-connectivity of the posterior cingulate cortex (*P* < 0.05). Specifically, in the LGI1 patient group, connectivity from the hippocampus to the superior temporal gyrus switched from excitatory to inhibitory, while connectivity from the superior temporal gyrus to the hippocampus shifted from inhibitory to excitatory. Furthermore, the inhibitory self-connection within the posterior cingulate cortex observed in controls was not seen in patients.

### Correlation analysis

**Figure 5:**
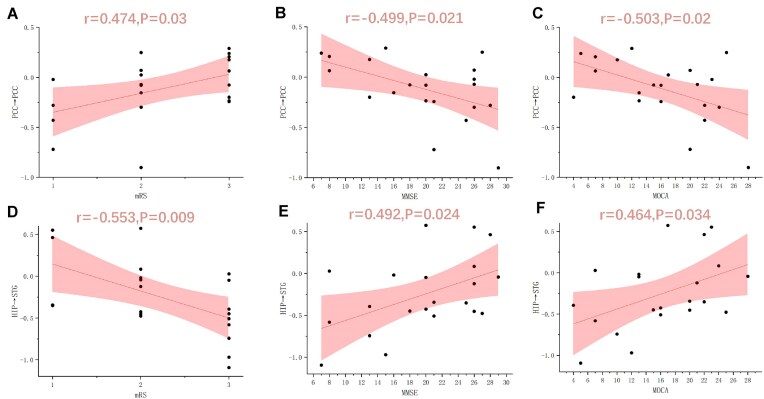
Correlation analysis results. (**A, B, C**) Correlation between PCC autoconnection strength and clinical mRS, MMSE, and MOCA scores, respectively. (**D, E, F**) Correlation between effective connection strength from the hippocampus to the superior temporal gyrus and mRS, MMSE, and MOCA scores, respectively.

Pearson correlations were computed between clinical scores and measures of effective connectivity to evaluate the relationship between clinical scales and connectivity characteristics. Figure 5 shows significant differences in mRS, MMSE, and MOCA scores between patients and controls. Notably, stronger posterior cingulate cortex self-connectivity in patients correlated positively with mRS scores (higher disability) and negatively with MMSE/MoCA scores (worse cognition). In contrast, the effective connectivity strength from the superior temporal gyrus to the hippocampus was negatively correlated with the mRS score and positively correlated with the MMSE and MOCA test scores (*P* < 0.05), indicating that stronger connectivity between these regions may be associated with better clinical outcomes and cognitive performance.

## Discussion

In this study, we investigated abnormalities in effective connectivity in anti-LGI1 encephalitis patients using spDCM and low-frequency oscillation amplitude analysis. Our findings revealed significant changes in the patient group in bidirectional effective connectivity patterns between the superior temporal gyrus and hippocampus and in self-connectivity patterns within the posterior cingulate cortex. Furthermore, the effective connectivity from the superior temporal gyrus to the hippocampus was correlated with clinical severity and cognitive performance scores, suggesting that these distinct causal connections might underlie neural dysfunction and potentially serve as biomarkers for anti-LGI1 encephalitis.

The LGI1 antibody is a secreted protein that enhances glutamatergic transmission and promotes neuronal excitation (Kornau *et al*., 2020). Previous research has documented structural abnormalities in the hippocampus and temporal lobe among anti-LGI1 encephalitis patients (Wang *et al*., 2017). Consistent with these observations, our study uncovered bidirectional changes in effective connectivity between the superior temporal gyrus and hippocampus in LGI1 encephalitis patients compared to healthy controls.

The superior temporal gyrus is crucial for auditory processing, short-term verbal memory, and social cognition (Bigler *et al*., 2007). Notably, anti-LGI1 encephalitis patients frequently experience epileptic seizures, often originating in the temporal lobe (Navarro *et al*., 2016). Furthermore, recent studies have reported hyperintensities in the medial temporal lobe, particularly among patients with facial tonic seizures and cognitive deficits (Wu *et al*., 2021). Additionally, ^18^F-FDG PET/computed tomography (CT) studies have demonstrated hypometabolism in the medial temporal lobe, suggesting that metabolic disturbances and changes in the temporal gyrus might contribute to the onset of LGI1 encephalitis (Liang *et al*., 2023; Roman *et al*., 2024).

The hippocampus is a vital component of the limbic system. It is responsible for transforming short-term into long-term memory and is integral to spatial memory formation (Lisman *et al*., 2017; Voss *et al*., 2017). Previous studies have documented hippocampal atrophy and impaired microstructural integrity in anti-LGI1 encephalitis (Finke *et al*., 2017; Szots *et al*., 2017; Heine *et al*., 2018; Hanert *et al*., 2019). Our previous research also revealed reduced functional connectivity within the hippocampus in this patient population (Qiao *et al*., 2020).

In the present study, we found that patients exhibited increased and excitatory effective connectivity from the superior temporal gyrus to the hippocampus, coupled with decreased connectivity in the reverse direction. These findings suggest an imbalance in information flow between these regions, potentially contributing to the clinical manifestations and cognitive deficits observed in anti-LGI1 encephalitis.

The posterior cingulate cortex is a pivotal component of the default mode network and serves as a central hub for facilitating information integration from diverse brain regions (Friston *et al*., 2008; Miao *et al*., 2011; Yan *et al*., 2013). Disruption of the posterior cingulate cortex has been implicated as a potential contributor to psychiatric manifestations, including emotional distress and cognitive deficits, in encephalitis patients (Sun *et al*., 2023). Studies utilizing electroencephalography (EEG) have highlighted posterior cingulate cortex's modulation of the DMN during task-negative states, revealing changes in its effective connectivity (Wang et al., 2019).

Consistent with previous research on anti-LGI1 encephalitis, which demonstrated enhanced functional connectivity within the DMN (Heine *et al*., 2018), our findings indicated a loss of inhibitory self-connectivity in the posterior cingulate cortex and an overall increase in connectivity strength among patients compared to controls. This altered pattern might lead to impairments in regulating networks essential for cognition, memory, and motor functions.

Our analysis revealed that the effective connectivity from the hippocampus to the superior temporal gyrus was negatively correlated with mRS scores, suggesting disease severity and was positively correlated with MMSE and MOCA scores, which are markers of cognitive function. Notably, the altered strength of self-connectivity within the posterior cingulate cortex was also found to correlate with clinical symptoms, displaying a positive correlation with mRS scores and a negative correlation with MMSE and MOCA scores. These observations suggest that the disruption of self-connection inhibition in the posterior cingulate cortex might be associated with impaired cognitive control in anti-LGI1 encephalitis. This finding could impair the ability to shift between cognitive states, leading to uncontrolled attention focus.

This study had several limitations. First, the relatively small sample size might limit the generalizability of our findings. Rare diseases usually necessitate multicenter collaborations to achieve adequate sample sizes to valid its homogeneity of characterization. So our results should be interpreted as preliminary until validated in larger, independent cohorts. Second, our study only focuses on a vertical assessment but longitudinal research is also vital, such as long-term cognitive trajectories or treatment responsiveness, which are critical for understanding disease progression. Thus future studies with extended follow-up periods are essential to confirm these findings. Third, our effective connectivity analysis was confined to four ROIs selected based on the amplitude of low-frequency fluctuations. Future studies should consider exploring the modulatory mechanisms of a broader range of brain networks. Last, while our current approach was important, the integration of multimodal imaging techniques, including diffusion tensor imaging, EEG, and structural MRI, will be crucial to obtaining a more comprehensive understanding of brain activity and the pathological neural mechanisms underlying this disorder.

In this study, we employed spDCM methods to examine the effective connectivity patterns in anti-LGI1 encephalitis patients. Our findings revealed inhibitory connections from the hippocampus to the superior temporal gyrus in these patients, exhibiting a negative correlation with disease severity and a positive correlation with cognitive function. Additionally, we observed increased effective connectivity within the posterior cingulate cortex in the patient group that was positively correlated with mRS scores. These alterations in causal interactions across distinct brain regions provide evidence for pathological neural deficits in anti-LGI1 encephalitis and may serve as potential biomarkers for personalized diagnosis and treatment strategies.
